# Assessing the Role of Inflammatory Markers in Predicting Central Lymph Node Metastasis in Papillary Thyroid Carcinoma

**DOI:** 10.22038/ijorl.2025.84332.3840

**Published:** 2025

**Authors:** Ebrahim Farashi, Sajjad Ranjbar, Sahand Eslami, Nikzad Shahidi, Seyed Ziaeddin Rasihashemi

**Affiliations:** 1 *Department of Otorhinolaryngology-Head and Neck Surgery, Tabriz University of Medical Sciences, Tabriz, Iran.*; 2 *Department of Pediatric Surgery, Tabriz University of Medical Sciences, Tabriz, Iran.*; 3 *Department of Cardiothoracic Surgery, Tabriz University of Medical Sciences, Tabriz, Iran.*

**Keywords:** Thyroid Nodule, Papillary Thyroid Carcinoma, Lymph Node, NLR, PLR

## Abstract

**Introduction:**

The current study sought to assess the predictive value of inflammatory markers, namely the platelet-to-lymphocyte ratio (PLR) and neutrophil-to-lymphocyte ratio (NLR), in relation to central lymph node metastasis (CLNM) in patients with papillary thyroid carcinoma (PTC).

**Materials and Methods:**

A total of 79 participants with confirmed PTC diagnosis following surgery were included in the study. Demographic data, clinical characteristics, and laboratory variables were collected. CLNM was evaluated based on pathological findings. NLR and PLR were calculated to assess their associations with clinicopathological features and metastasis.

**Results:**

The study included 26 males (25.9%) and 53 females (74.1%), with a mean age of 42.26 years (range 15–74). Significant findings included a higher incidence of CLNM in males (*p*=0.047) and larger tumor sizes in patients with CLNM (*p*=0.047). Tumor multifocality was also more common in the CLNM group (62.5% vs. 28.9%, *p*=0.003). Although no significant relationship was found between the inflammatory markers NLR, PLR, and CLNM, a meaningful association was observed between PLR and age in patients with CLNM (*r*= 0.414, *p*=0.011).

**Conclusion:**

This study demonstrates significant associations between clinicopathological features and CLNM in PTC. Male gender, larger tumor size, and multifocality correlated with higher CLNM rates. While inflammatory markers such as NLR and PLR did not show significant differences, PLR's positive correlation with age in CLNM patients suggests potential prognostic value, warranting further investigation.

## Introduction

The most prevalent endocrine condition is thyroid nodules, which are defined by the thyroid cells' aberrant development that results in a lump in the thyroid gland. Typically, these nodules are asymptomatic and discovered during physical examinations or incidentally through imaging studies. While most nodules are benign, 3–7% are malignant (1). About 80% of instances of thyroid cancer are papillary thyroid carcinoma (PTC). When assessing thyroid nodules, fine needle aspiration (FNA) is the main diagnostic method (2). Predicting malignancy in neoplastic nodules has been the focus of numerous studies, which have examined ultrasonographic and pathological features (1,3-5).Numerous malignancies, including those of the esophagus, stomach, pancreas, colon, ovary, kidney, and lung, have been investigated for their ability to be predicted by inflammatory markers such as tumor necrosis factor-alpha as well as C-reactive protein, interleukin-6, and the neutrophil-to-lymphocyte ratio (NLR) (6-8). The relationship between inflammation and thyroid cancer has been established (9,10), with markers like NLR and the platelet-to-lymphocyte ratio (PLR) gaining attention (11-13). These indicators' use in the preoperative assessment of PTC is still restricted and debatable, nevertheless. This study aimed to assess the predictive power of the inflammatory markers NLR and PLR in predicting central lymph node metastases in patients with papillary thyroid carcinoma. 

## Materials and Methods 

*Study Design and Participants*

In this cross-sectional descriptive analysis, 79 individuals with post-operative PTC diagnoses between the ages of 18 and 65 were included. Between March 2020 and March 2021, all patients at Imam Reza Hospital, which is connected to Tabriz University of Medical Sciences in Tabriz, Iran, had a complete thyroidectomy and central lymph node dissection. The patients were divided into two groups—those with CLNM and those without—based on postoperative pathology. Infections or hematological disorders, other cancers, head and neck radiation, coronary heart disease, autoimmune diseases, lymphocytic infiltration indicating thyroiditis in histopathology, liver or kidney dysfunction, prior thyroid surgery, or chronic drug use (e.g., steroids) were all reasons for exclusion. Demographic data, including sex, age, BMI, and medical history, were extracted from the patients' medical records. 

### Clinicopathological Evaluation 

Clinicopathological evaluations were conducted to assess tumor size, multifocality, and the presence of CLNM. The frequency and impact of CLNM in PTC patients were analyzed, and clinicopathological differences between those with and without CLNM were statistically evaluated. Nodules were documented as either solitary (unifocal) or multiple (multifocal), and their status regarding CLNM was recorded. 

### Laboratory and Inflammatory Marker Analysis 

Preoperative blood samples were analyzed for complete blood count parameters, including white blood cells (WBC), lymphocytes as well as monocytes, neutrophils and platelets. NLR and PLR were calculated for each patient and compared between PTC and non-PTC groups, as well as between patients with and without CLNM, to evaluate their predictive value for tumor malignancy and metastasis. 

### Statistical Analysis 

For data processing, version 22 of IBM SPSS Statistics was utilized. The Kolmogorov-Smirnov test was further employed to determine the normality distributions for quantitative data. On the other hand, percentages and frequencies were used to present qualitative data. In the instance where quantitative data was normally distributed, the mean and standard deviation were reported while for skewed data, median (25th and 75th percentiles) values were communicated. The chi-square test or Fisher’s exact test (when applicability of the chi-square test was not possible) were used for qualitative data. At the same time, for quantitative data that involved comparison of two groups, the Mann-Whitney test was applied for non-normally distributed data whereas independent t-test was used for normally distributed data. Any p-value that was below 0.05 was considered statistically significant.

## Results

### Patient Demographics and Clinical Characteristics 

The study included 79 participants with confirmed PTC diagnosis after surgery. Demographic data, clinical characteristics, and laboratory variables were collected. CLNM was evaluated based on pathological findings, and NLR and PLR were calculated to explore their associations with clinicopathological features and metastasis. The participants comprised 26 males (32.91%) and 53 females (67.09%), with a mean age of 42.25 years (±13.97). The demographic and clinical variables of the patients are summarized in Table 1.

**Table 1:** Demographic status and clinical and laboratory variables of the patient.

Variable	Frequency (Interquartile domain) n=79
Age	42.25 ± 13.97
Gender Male Female	26 [32.91%] 53 [67.09%]
BMI	25 (22_28)
WBC	6.85 (5.62_8.9)
Neutrophil	4.05 (2.89_5.25)
Lymphocyte	1.6 ± 2.58
Platelet	237.682 ± 61.000
PLR*	108 (86.62_149.82)
NLR**	1.94 (1.38_3.1)
Tumor size (n=80)	2.25 (1.2_3.57)
CLNM*** Yes No	41 [51.9%] 38 [48.1%]
Multifocality (n=78) Multifocal (n=36) Unifocal (n+42)	36 [45.5%] 42 [54.5%]

*PLR: Platelet to Lymphocyte ratio, **NLR: Neutrophil to Lymphocyte ratio, ***CLNM: Central Lymph node metastasis

### Comparison of CLNM and Non-CLNM Patients 

The age distribution of patients with and without CLNM did not differ significantly (*p>*0.05). 

Nonetheless, there was a significant correlation between gender and CLNM, with a greater occurrence among male patients (*p=*0.047). The median NLR was slightly higher in patients with CLNM, and the median PLR was also elevated, but neither difference reached statistical significance (*p*>0.05). Patients with CLNM had noticeably bigger tumors (*p*=0.047), and multifocality was more frequent in this group (62.5% vs. 28.9%, *p*=0.003). CLNM was observed in 51.9% of patients with PTC, while no non-PTC patients exhibited CLNM (Table 2). 

**Table 2:** Clinicopathological Characteristics of patients with and without CLNM

**Variable**	**Patients with CLNM** **Average frequency (Interquartile domain) n=41**	**Patients without CLNM** **average frequency (Interquartile domain) n=38**
Age	42.24 ± 15.39	42.27 ± 12.25
Gender Male Female	16 [61.5%] 25 [47.1%]	10 [38.5%] 28 [53.9%]
BMI	25 (22-28)	25 (23-27)
WBC	7.2 (6-8.9)	6.5 (5.62-8.27)
Neutrophil	4.18 (3.35-5.25)	3.93 (2.89-5.19)
Lymphocyte	1.99 (1.6-2.49)	2.17 (1.69-2.58)
Platelet	228487 ± 61949	245808 ± 60090
PLR	112.94 (86.62-144.49)	104.16 (87.50-149.82)
NLR	1.92 (1.56_3.1)	1.96 (1.38-2.37)
Tumor size (n=80)	2.5 (1.7-3.57)	2 (1.2-2.67)
Multifocality (n=78) Multifocal Unifocal	25 [62.5%] 15 [37.5%]	11 [28.9%] 27 [71.1%]

* r: Spearman's correlation coefficient

### Relationship Between NLR, PLR, and Clinicopathological Features 

No significant associations were observed between NLR or PLR and age, gender, tumor size, or multifocality in the overall patient population (*p*>0.05). 

**Fig 1:**
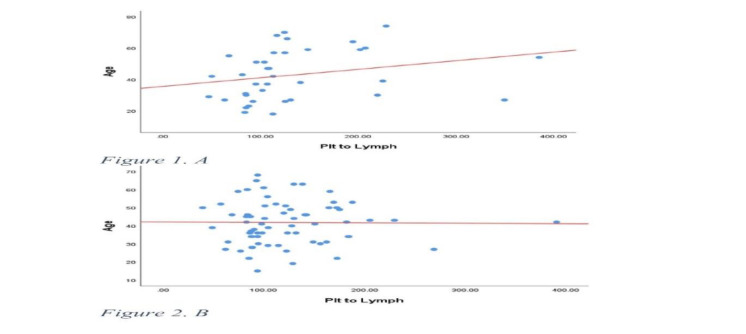
A**: **PLR and age relation in patients with CLNM _ B: PLR and age relation in patients without CLNM

However, a significant positive correlation was identified between PLR and age in patients with CLNM (*r*=0.414, *p*=0.011) (Figure 1). No significant association was found between PLR and tumor size in patients with CLNM (*p*=0.862, *r*=0.030). Additionally, no significant associations were identified between NLR or PLR and clinicopathological factors in patients without CLNM (*p*>0.05) (Table 3). 

**Table 3:** The correlation between PLR and NLR by study group, age, gender, tumor size, and multifocality

**Patient group**	**Variable**		**PLR**	**NLR**
All of the patients	Age	r*	0.191	0.055
		p-value	0.055	0.583
	Tumor size	r	0.096	0.106
		p-value	0.435	0.389
	Gender	r	0.214	0.125
		p-value	0.069	0.225
	Multifocality	r	0.217	0.336
		p-value	0.075	0.511
With CLNM	Age	r	0.414	0.179
		p-value	0.011	0.289
	Tumor size	r	0.030	0.054
		p-value	0.862	0.754
	Gender	r	0.300	0.114
		p-value	0.163	0.183
	Multifocality	r	0.098	0.236
		p-value	0.521	0.214
Without CLNM	Age	r	0.045	-0.014
		p-value	0.724	0.913
	Tumor size	r	0.112	0.058
		p-value	0.542	0.754
	Gender	r	0.885	0.749
		p-value	0.658	0.714
	Multifocality	r	0.654	0.253
		p-value	0.125	0.332

## Discussion

This study sought to determine how preoperative NLR and PLR can predict CLNM in PTC patients. The median NLR was somewhat greater for patients with CLNM than for those without, but the difference was not statistically significant. Likewise, although PLR levels in the CLNM group were greater, they fell short of statistical significance. Our study revealed that male gender, larger tumor size, and multifocality were significantly associated with a higher incidence of CLNM in PTC patients. The significant association of gender with CLNM suggests that male patients may have a higher risk for metastasis, which could guide clinical decision-making. Multifocality may indicate a greater likelihood of metastatic spread, emphasizing the need for close monitoring of patients with multifocal tumors for CLNM. 

These findings align with previous studies identifying male gender and larger tumor size as key risk factors for CLNM in PTC (14–17) and with studies highlighting the significance of multifocality in thyroid cancer prognosis (14-16). Early research has shown that elevated NLR levels are linked to larger tumors and an increased risk of recurrence, according to the American Thyroid Association classification. This suggests that patients with higher NLR may face a greater likelihood of recurrence and worse prognosis (17). However, other studies have not found these associations but suggest that higher NLR levels predict a poorer histopathological profile and more aggressive clinical behavior (18). 

For example, Feng et al. demonstrated that pretreatment NLR could serve as a robust biomarker for predicting lymph node metastasis in thyroid cancer (19). Similarly, Özdemir et al., in a retrospective study of 441 thyroidectomy cases, suggested that NLR might be valuable in detecting lymph node metastasis in PTC (20). Furthermore, Chen et al. identified high pretreatment NLR as a significant risk factor for extensive lymph node metastases in PTC (21). However, in line with our findings, several other studies reported no significant association between NLR and the prediction of PTC metastasis (22,23).

Cao et al. found that higher PLR values were associated with more advanced lymph node (N) stages, larger tumors, and poorer clinicopathological features in PTC (23). Similarly, Chen et al. developed a predictive model indicating that elevated platelet levels are associated with lymph node metastasis in PTC (21). 

Wang et al., in a retrospective study of 117 differentiated thyroid cancer patients, also identified platelets as useful predictors of thyroid cancer malignancy and lymph node metastasis (24). Ari et al. observed elevated NLR and PLR levels in patients with thyroiditis and PTC but concluded that PLR was not useful for distinguishing between benign and malignant conditions (25). Consistent with our findings, Zhao et al. reported that neither NLR nor PLR significantly impacted lymph node metastasis in PTC patients (26). Additionally, Özdemir et al. noted that while PLR had some correlation with lymph node metastasis detection, its predictive value was limited (20). 

In our study, no significant correlations were found between NLR or PLR and age, gender, tumor size, or multifocality in the overall patient population. However, a significant positive correlation between PLR and age was observed in patients with CLNM, suggesting that PLR tends to increase with age in this subgroup. This finding may have implications for understanding age-related immune responses in PTC. 

Multifocality, likewise, indicates a higher likelihood of metastatic spread, underscoring the importance of vigilant monitoring in patients with multifocal tumors. 

The lack of significant relationships in our study may be attributed to the heterogeneity of PTC (27) and the influence of other clinical and molecular factors, such as the BRAF V600E mutation, which is associated with a pro-inflammatory state and higher TNM stages (28). These factors can affect inflammatory responses and immune status (9,29). Additionally, the tumor microenvironment and immune cell infiltration may play roles in PTC progression (34). 

These diverse findings suggest that the utility of NLR and PLR in predicting central lymph node metastasis and tumor aggressiveness may depend on patient demographics, tumor characteristics, and other factors. Furthermore, confounding factors such as diabetes, smoking, inflammatory diseases, iron deficiency, and the use of antiplatelet medications, which can influence platelet indices, were not considered in our study (30-32). 

Further research is required to clarify the relationship between inflammatory markers like NLR and PLR and the development of central lymph node metastasis and tumor invasion in PTC. Large, prospective multicenter studies are needed to evaluate these biomarkers and establish their clinical relevance in managing PTC. 

## Conclusion 

This study identifies significant associations between clinicopathological features and CLNM in PTC. Male gender as well as larger tumor size and multifocality were associated with higher CLNM rates. Although inflammatory markers such as NLR and PLR did not show significant differences, the positive correlation between PLR and age in CLNM patients suggests potential prognostic value that warrants further investigation.
